# Familial Near-Total Intestinal Aganglionosis

**DOI:** 10.21699/jns.v6i3.562

**Published:** 2017-08-10

**Authors:** Hidouri Saida, Zitouni Hayet, Chahed Jamila, Mosbahi Sana, Belhassen Samia, Ksiaa Amine, Hmida Badii, Krichene Imed, Sahnoun Lassad, Mekki Mongi, Belguith Mohsen, Nouri Abdellatif

**Affiliations:** 1 Department of Pediatric Surgery, Medical School Hospital of Monastir, Tunisia; 2 Department of Radiology, Medical School Hospital of Monastir, Tunisia; 3 Research Laboratory LR12SP13. Faculty of Medicine of Monastir, Tunisia

**Keywords:** Hirschsprung’s disease, Total intestinal aganglionosis, Genetic, Familial

## Abstract

Near total aganglionosis represents the most extreme and rare form of Hirschsprung's disease. It can affect more than one member of family. We report three cases of near total intestinal aganglionosis in a family presenting with intestinal obstruction at birth. All of them were operated and a jejunostomy was performed. Outcome was dismal.

## INTRODUCTION

Absence of ganglionic innervations throughout the entire gastrointestinal tract represents the most extreme and rare form of Hirschsprung Disease (HD). It occurs in about 3% to 5% of HD cases [1]. HD affects more than one member of the family in 4-8 % of cases. [2]. Herein we report three cases of near total HD within one family.

## CASE SERIES

**Case 1:**

The proband was a baby boy, born to a nonconsanguineous Tunisian couple after an uneventful pregnancy and normal labor. His birth weight was 3850g. Soon after birth, the infant presented with bilious vomiting and delayed passage of meconium. There was no family history of Hirschsprung disease. His older brother had a good health. Rectal examination was interpreted as normal. His abdominal plain x-ray at day 3 of life showed an intestinal obstruction. At laparotomy, all intestine beyond mid-ileum was collapsed without evidence of intrinsic obstruction. Appendectomy was performed. Pathologic examination of the appendix confirmed the diagnosis of Hirschsprung disease. An ileostomy constructed 80 cm distal to the ligament of Treitz did not function. On the seventh postoperative day, the infant had undergone abdominal re-exploration with replacement of the ileostomy by a proximal jejunostomy. The stoma still not worked and died at 4 weeks of age. Biopsy specimens of both ileostomy and jejunostomy stained with Hematoxylin and Eosin showed aganglionosis.

**Case 2:**

A second sibling of the same family, was a full-term male newborn with birth weight of 3200g. A prenatal scan showed intestine dilatation. At first day of life, the newborn presented with vomiting with abdominal distension. Abdominal plain x-ray showed an intestinal obstruction. Barium enema showed a micro colon. On day 6 of life laparotomy showed a dilated intestine (extended to 30 cm from the ligament of Treitz). Frozen sections done on full thickness biopsies of appendix, mid-ileum, mid-jejunum and the antrum demonstrated an absence of ganglion cells. A jejunostomy 20 cm distal to the ligament of Treitz was performed. Post-operatively, the jejunostomy did not function and the infant died at day 11 of life.

**Case 3:**

The fourth child to this couple was a full-term female, born by a cesarean delivery with a birth weight of 2700g. The newborn did not pass meconium within the first 48 hours after birth followed by bilious vomiting. On plain abdominal x-ray, air fluid levels were noted. Barium enema showed a micro colon. A diagnosis of Hirschsprung disease was made based on the family history of near total aganglionosis, as well as the clinical and radiological manifestations. At laparotomy, the entire intestine was collapsed (Fig.1). Multiples biopsies from the stomach to the colon demonstrated no ganglion cells. The patient died of intestinal obstruction at day 24 of life.

**Figure:**
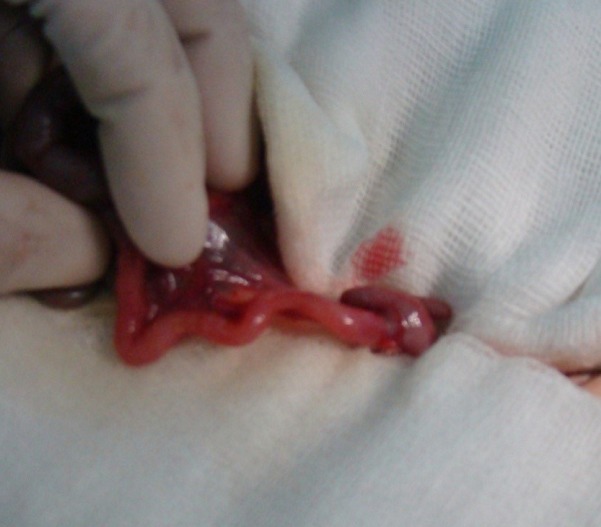
Figure 1 : Collapsed intestine.

## DISCUSSION

Aganglionosis involving most of the bowel has the highest morbidity and mortality rate due to persistent intestinal obstruction, malnutrition and infections [3]. Long segment HD has familial tendencies. Affected families are known to carry as high as 200 times higher risk of recurrences [4]. It is generally accepted that in long HD, the inheritance pattern is autosomal dominant with incomplete penetrance. Other reports suggest autosomal recessive transmission. RET proto-oncogene variations were identified in 70% of the familial group vs 30% of non-familial cases, but no specific sites in the gene were identified [3]. In our study, no genetic test was carried out.

Management of patients with near total aganglionosis is a challenge. Although significant progress in parenteral nutrition is achieved, mortality rate is considerably high. Certain procedures have been recommended such as Ziegler myectomy, serial tapering enteroplasty, and intestinal transplantation as a curative approach [5–7]. In literature, the mean age of survival in near total HD ranges from 7 days to 5 years [5]. In our experience, the mean age of survival was 21 days.

In conclusion, near total intestinal aganglionosis with absence of ganglion cells from the stomach, duodenum to the rectum is a rare form of HD. Outcome is dismal without latest treatment modalities such as intestinal transplantation.

## Footnotes

**Source of Support:** None

**Conflict of Interest:** None
